# Beyond VO_2max_: Integrated Physiological Phenotyping and Candidate-Gene Analysis of Elite Versus Recreational Endurance Runners

**DOI:** 10.3390/genes17091133

**Published:** 2026-09-17

**Authors:** Hasan Uğur Öncel, Jason Siegler, Alper Özkan, Okan Oğul, Mikail Yalçın, Şahabettin Karabulut, Rıfat Gülmez, Seyed Houtan Shahidi

**Affiliations:** 1 Department of Nutrition and Dietetics, Faculty of Health Sciences, Istanbul Gedik University, Kartal, Istanbul 34876, Türkiye; dytokanogul@gmail.com; 2 Integrative Human Performance Lab, College of Health Solutions, Arizona State University, Phoenix, AZ 85004, USA; jason.siegler@asu.edu; 3 Department of Cardiology, Acıbadem Hospital, Istanbul 34149, Türkiye; alper.ozkan@gedik.edu.tr; 4 Department of Sports Coaching, Faculty of Sports Sciences, Istanbul Gedik University, Istanbul 34906, Türkiye; mikailyalcin65@gmail.com (M.Y.); sahabettin2109@gmail.com (Ş.K.); rifattgulmez@gmail.com (R.G.); houtan.shahidi@gedik.edu.tr (S.H.S.)

**Keywords:** endurance running, maximal oxygen uptake, lactate threshold, running economy, dual-energy X-ray absorptiometry, ACTN3, VEGFA, PPARGC1A, ACE, PPARG, principal component analysis, sports genomics

## Abstract

**Background:** Endurance performance reflects integrated cardiorespiratory, metabolic and musculoskeletal traits with substantial heritability, yet sex-balanced studies that simultaneously deep-phenotype athletes and genotype candidate loci against trained comparators remain scarce. **Objective:** We aim to characterize the integrated physiological profile and genotype distributions at five endurance-relevant polymorphisms in elite versus recreationally trained runners. **Methods:** Eighty runners (20 elite male, 20 elite female, 20 recreational male, 20 recreational female) completed graded treadmill testing with breath-by-breath gas exchange and capillary blood lactate sampling, together with whole-body dual-energy X-ray absorptiometry. A sub-sample of 40 participants (10 per subgroup) underwent next-generation sequencing of *ACTN3* R577X, *VEGFA* rs699947, *PPARGC1A* Gly482Ser, *ACE* I/D and *PPARG* Pro12Ala. Physiology was compared using one-way ANOVA with Tukey’s HSD and Cohen’s d; genotype distributions were compared using Monte Carlo exact tests with Benjamini–Hochberg correction. **Results:** Elite runners exhibited higher VO2max (elite male 71.8 ± 4.9; elite female 65.3 ± 3.6 mL·kg^−1^·min^−1^; both vs. same-sex controls, *p* < 0.001; η^2^ = 0.62), superior running economy (η^2^ = 0.76), lactate thresholds shifted rightward by ~1 km·h^−1^, and more favorable body composition (lean mass η^2^ = 0.80; body fat η^2^ = 0.48). Principal component analysis separated elites from recreational runners along PC1 (40.1% variance) in both sexes. Effect-allele frequencies were numerically higher in elites at every locus (odds ratios 1.48–2.03), but no genotype × group association survived correction (all q ≥ 0.82). **Conclusions:** Elite endurance status was robustly distinguished by an integrated physiological phenotype with large effect sizes. Effect-allele frequencies at five candidate loci were numerically higher in the expected direction but did not reach statistical significance; given the small genotyped sub-sample, these genetic data are purely exploratory and hypothesis-generating and require replication in adequately powered cohorts.

## 1. Introduction

Endurance running performance is the paradigmatic complex phenotype in exercise physiology, emerging from the coordinated action of oxygen delivery, substrate metabolism, and neuromechanical efficiency [1]. A century of laboratory science has established maximal oxygen uptake (VO2max) as the ceiling of aerobic capacity and a strong correlate of competitive success [2]. VO_2_max alone, however, explains only a fraction of the between-athlete variance in elite race performance [3]. Two further physiological pillars, the fractional utilization of VO2max at which blood lactate accumulates (the lactate threshold) and the oxygen cost of submaximal running (running economy), account for much of the residual variance and often distinguish world-class runners from merely well-trained runners [4]. Body composition further modulates this triad: low relative fat mass, high lean mass, and adequate bone mineral density lower the metabolic cost of transport and preserve long-term training availability [5]. Genetics substantially influences each of these traits [6]. Twin and family designs estimate the heritability of VO2max at approximately 45–50%, with comparable or higher estimates for the training response [7]. Two decades of candidate-gene and, more recently, genome-wide association research have implicated specific polymorphisms in endurance-related phenotypes. Null homozygosity at ACTN3 (R577X, rs1815739) eliminates α-actinin-3 from fast-twitch muscle fibers and has been associated with a relative shift toward oxidative, slow-twitch phenotypes [8,9]. Promoter variation at VEGFA (including rs699947) modulates exercise-induced angiogenesis and skeletal-muscle capillarization [10]. The PPARGC1A Gly482Ser substitution (rs8192678) alters signaling through a master regulator of mitochondrial biogenesis [11]. The ACE insertion/deletion polymorphism influences circulating angiotensin-converting enzyme activity and has been linked to elite-endurance status across multiple cohorts, most consistently with the I allele [12]. The PPARG Pro12Ala variant (rs1801282) affects lipid handling and insulin sensitivity, although its contribution to elite endurance remains contested [13]. Despite a broad range of candidate-gene literature, three gaps limit the translational value of existing evidence. First, integrated datasets that combine deep physiological phenotyping (gas exchange, lactate kinetics, DEXA-derived body composition) with targeted genotyping in a single cohort are scarce; most studies provide either large athlete cohorts with coarse phenotypes or small, richly phenotyped samples without genetic data. Second, sedentary rather than trained comparators dominate the literature, inflating apparent effect sizes and obscuring what actually distinguishes elite runners from the well-trained. Third, female athletes remain substantially underrepresented, leaving sex-stratified inference underdeveloped [14]. Addressing these gaps requires sex-balanced, simultaneously phenotyped and genotyped samples compared with trained comparators. The present pilot study integrates graded treadmill testing, DEXA, and targeted next-generation sequencing of five canonical endurance loci in a sex-balanced sample of elite and recreationally trained runners. We tested three hypotheses: (i) elite runners would demonstrate superior VO2max, running economy, lactate thresholds, and body composition relative to recreational counterparts; (ii) alleles reported as endurance-favorable in the published literature would be over-represented in elite runners; and (iii) multivariate projection of the physiological variables would yield a low-dimensional latent structure that discriminates elite from recreational phenotypes in both sexes.

## 2. Materials and Methods

### 2.1. Study Design and Ethical Approval

This was a cross-sectional case–control investigation. The protocol was approved by the Ethics Committee of Istanbul Gedik University (approval number E-56365223-050.04-2025.137548.39) and complied with the Declaration of Helsinki (2013 revision). Written informed consent was obtained from every participant before any procedure.

### 2.2. Participants and a Priori Sample Size

An a priori sample-size calculation (G*Power 3.1) for an independent-samples *t*-test (two-tailed; α = 0.05; power = 0.80; Cohen’s *d* = 0.80, corresponding to reported VO2max differences between elite and recreational runners) returned a minimum of 26 participants per group. We recruited 40 participants per group (n = 80 total), yielding >95% power for the primary VO2max contrast. For the genetic sub-study (10 per cell; n = 40 in total), equivalent assumptions detect only very large allele-frequency shifts (Δf ≈ 0.30); this component is therefore explicitly underpowered for single-locus inference and is framed as exploratory throughout [15].

**Figure 1:**
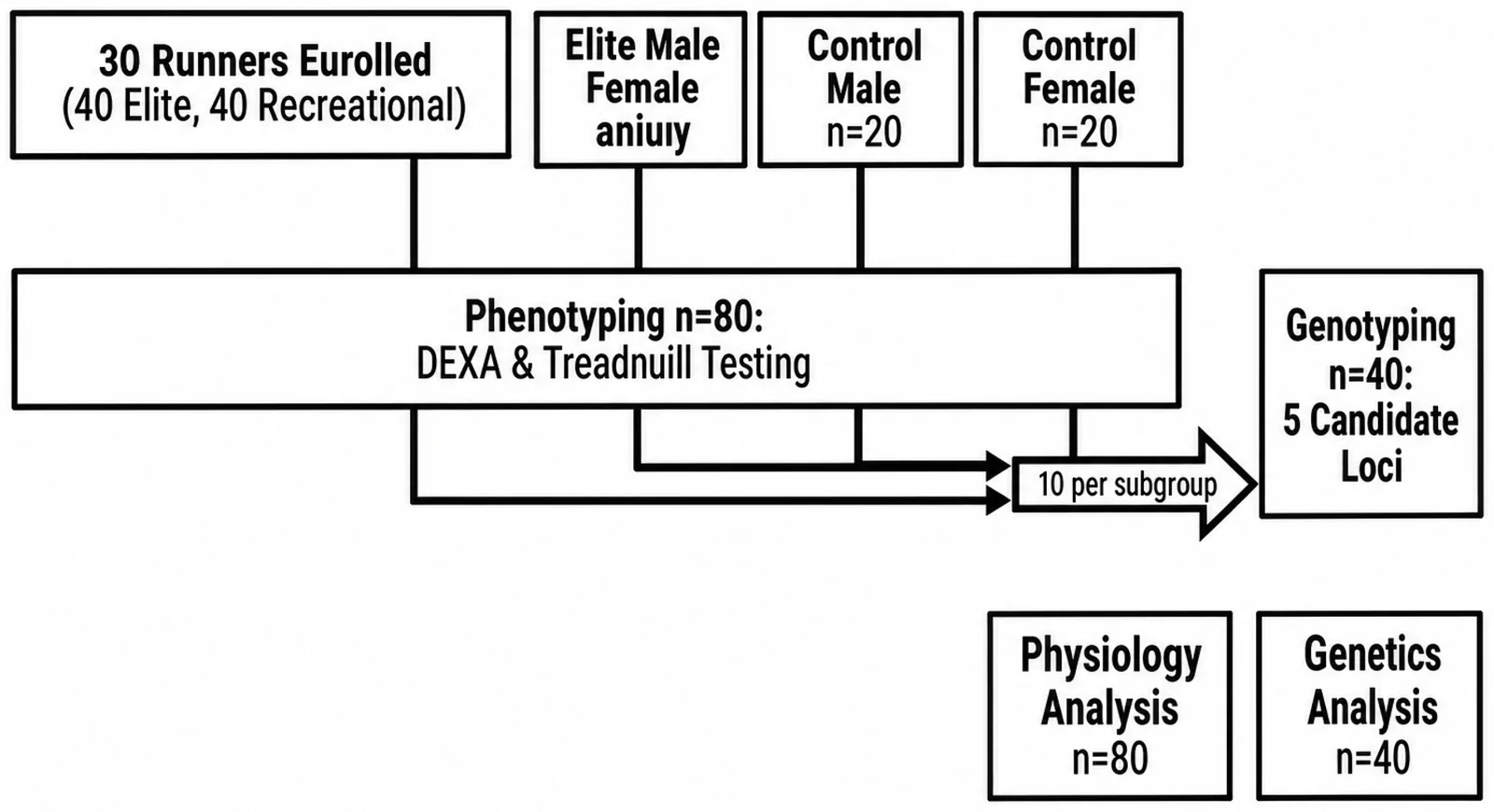
Schematic representation of the data analysis workflow.

*The elite group* (n = 40; 20 males, 20 females; 27.4 ± 2.9 yr) comprised competitive middle- and long-distance runners (3000 m steeplechase, 5000 m, 10,000 m, half-marathon or marathon) with ≥10 years of structured training and international-level competitive experience. Personal-best times were independently verified against the World Athletics database. *The recreational control group* (n = 40; 20 males, 20 females; 27.8 ± 3.4 yr) comprised runners with ≥4 years of habitual training (3–5 weekly sessions of 45–75 min; 5–10 km) who had never competed at national or international level. Exclusion criteria for both groups were cardiopulmonary or metabolic disease, musculoskeletal injury within the preceding three months, regular medication affecting cardiovascular or metabolic variables, and pregnancy (Figure 1).

### 2.3. Body Composition

Whole-body dual-energy X-ray absorptiometry scans were performed on a GE Lunar iDXA densitometer (GE Healthcare, Madison, WI, USA) at Acıbadem Altunizade Hospital in Istanbul, Turkey. Participants attended after an overnight fast, refrained from strenuous exercise for 24 h, and were scanned in light clothing with all metal removed. Fat mass, lean soft-tissue mass, and total and regional bone mineral content and density were quantified using enCORE software version 17. The scanner was calibrated daily against the manufacturer’s phantom; the in-house coefficient of variation for repeated scans was <1.0% for lean mass and <1.5% for fat mass. Operators were blinded to group allocation.

### 2.4. Graded Treadmill Protocol

All testing was performed on a Trackmaster TMX428 motorized treadmill (Full Vision Inc., Newton, KS, USA; set at a 1% gradient) in a climate-controlled laboratory (20–22 °C; 45–55% relative humidity). Treadmill speed was automatically controlled by the MetaMax 3B portable breath-by-breath metabolic analyzer (Cortex Biophysik GmbH, Leipzig, Germany) via a dedicated RS-232 serial interface, with the preprogrammed protocol executed directly from the MetaSoft Studio control software (version 5.2.1; Cortex Biophysik GmbH, Leipzig, Germany). Rather than stepped transitions, the MetaMax issued continuous micro-adjustment commands every 4 s, so treadmill velocity increased smoothly at a net rate of 1 km·h^−1^ per minute throughout the test. This arrangement eliminated manual speed changes and transient artifacts associated with operator-driven protocols, ensuring precise temporal alignment between gas-exchange samples and running velocity. The MetaMax 3B analyzer was calibrated before every test following the manufacturer’s three-stage procedure. First, a volume calibration was performed using a 3-L precision syringe (Hans Rudolph Inc., Shawnee, KS, USA), with five complete strokes delivered across low, medium, and high flow rates; calibration was accepted only when the mean volume deviation remained within ±2.0%. Second, a reference-gas calibration was performed against a certified gas mixture (15.00% O_2_/5.00% CO_2_, balance N_2_; traceable to a national standard), used to set the span of both the paramagnetic O_2_ and infrared CO_2_ analyzers. Third, an ambient-air (zero) calibration was carried out in the laboratory environment, with local barometric pressure, temperature, and relative humidity entered manually into the MetaSoft interface. Any drift exceeding the manufacturer’s tolerance at any of the three stages triggered recalibration before testing proceeded [16].

Participants abstained from caffeine, strenuous exercise, and alcohol for 24 h and completed a standardized 10-min warm-up consisting of 5 min of easy running and 5 min of dynamic mobility drills. The protocol broadly followed the continuous-ramp approach of Jones and colleagues. Female participants began at 8 km·h^−1^ and males at 10 km·h^−1^; under automatic MetaMax–Trackmaster control, treadmill velocity then increased by 1 km·h^−1^ per minute until volitional exhaustion [17]. Capillary fingertip blood (20 µL) was sampled at the end of each 1-min speed increment and analyzed immediately on a Scout 4 lactate analyzer (EKF Diagnostics, Barleben, Germany), calibrated before each session against the manufacturer’s two-point standard. Heart rate was recorded continuously via a Polar H10 chest strap paired with the MetaMax.

Breath-by-breath gas exchange was sampled at the mouth with the MetaMax 3B, transmitted in real time to MetaSoft Studio along with synchronous treadmill-velocity telemetry, and averaged over 30-s epochs. VO2max was defined as the highest 30-s mean recorded during the final 45 s of the test, provided that a plateau was observed (Δ VO_2_ < 150 mL·min^−1^ between the last two 30-s windows) and at least one of the following: respiratory-exchange ratio ≥ 1.10, heart rate within ±10 bpm of the age-predicted maximum, or blood lactate ≥ 8 mmol·L^−1^ [18]. Running economy was expressed as the steady-state VO2 (L·min^−1^) at 13 km·h^−1^, averaged over the final 30 s of that 1-min window. Because the protocol employed a continuous ramp (1 km·h^−1^ per minute with 4-s micro-adjustments) rather than discrete constant-load stages, the VO2 value averaged over the final 30 s of each 1-min stage should be interpreted as a standardized quasi-steady-state reference rather than a classic steady state. In the moderate-intensity domain (below LT1), pulmonary VO2 kinetics in trained runners exhibit a phase-II time constant of approximately 20–30 s, such that VO2 approaches the amplitude corresponding to the imposed work rate within the final 30 s of each stage. Critically, the ramp rate, stage duration, and averaging window were identical for all participants; any residual kinetic lag was therefore systematic and equal across subgroups and could not bias between-group comparisons. This continuous-ramp approach follows the methodology of Jones and colleagues [17] and eliminates transient artifacts associated with operator-driven speed changes. The first (LT1) and second (LT2) lactate thresholds were identified individually from each participant’s lactate–speed curve using the Dmax method and the fixed 4 mmol·L^−1^ criterion, respectively, with visual verification in ExPhysLab [19].

### 2.5. Genotyping

Genomic DNA was collected using Oragene saliva kits (DNA Genotek, Ottawa, ON, Canada), stored at −20 °C, and shipped within 72 h to Agiomix Medical Laboratory (Dubai, United Arab Emirates; ISO 15189-accredited [20]). DNA was extracted using a QIAamp DNA Mini Kit (Qiagen, Hilden, Germany); concentration and purity were verified by NanoDrop spectrophotometry (A260/280 > 1.8) and agarose gel electrophoresis. Libraries were prepared with Nextera XT Illumina, San Diego, CA, USA)and sequenced on an Illumina MiSeq platform (150-bp paired-end) to a minimum mean coverage of 100× over target regions. Reads were aligned to GRCh38 with BWA-MEM; variants were called with GATK HaplotypeCaller following GATK best practices, annotated against dbSNP build 155, ClinVar, and the 1000 Genomes Project, and functionally classified with SIFT and PolyPhen-2. Five loci were analysed: *ACTN3* R577X (rs1815739; coded XX/RX/RR); *VEGFA* −2578 C/A (rs699947; CC/CA/AA); *PPARGC1A* Gly482Ser (rs8192678; Gly/Gly/Gly/Ser/Ser/Ser); *ACE* I/D (287-bp intronic indel; II/ID/DD); and *PPARG* Pro12Ala (rs1801282; CC/CG/GG). For each locus, the allele previously associated with endurance-favorable phenotypes, *ACTN3* 577X, *VEGFA* −2578C, *PPARGC1A* 482Gly, *ACE* I, and *PPARG* 12Pro, was designated the effect allele. The genetic sub-study was restricted to 40 of the 80 phenotyped participants (10 per subgroup), selected via stratified random sampling from the consented list within each subgroup. The genetic sub-study was restricted to 40 of the 80 phenotyped participants (10 per subgroup), selected via stratified random sampling from the consented list within each subgroup. This sub-sample size is constrained by the budget for next-generation sequencing; consequently, this component is explicitly under-powered for single-locus or genotype–phenotype inference and is framed throughout as strictly exploratory. Participant ancestry was self-reported and broadly homogeneous (single-region recruitment); no formal genetic ancestry control (e.g., ancestry-informative markers or genome-wide principal component analysis) was performed, and this is addressed explicitly in the Limitations.

### 2.6. Statistical Analysis

All statistical analyses were performed in OriginPro 2024 (OriginLab Corporation, Northampton, MA, USA). Continuous data are reported as mean ± SD with 95% confidence intervals (CIs). Normality and homogeneity of variance were assessed with Shapiro–Wilk and Levene’s tests, respectively. Given the balanced 2 × 2 factorial design, we examined between-group differences in single-timepoint physiological and body-composition variables using two-way ANOVA to evaluate the main effects of competitive status (elite vs. recreational), sex (male vs. female), and their interaction (status × sex). Welch’s ANOVA was used when the assumption of homogeneity of variance was violated. Where significant interactions were observed, simple main effects were analyzed with Tukey HSD post hoc comparisons.

Because running economy and blood lactate were measured across multiple incremental speeds within the same participants, these variables were analyzed using a two-way repeated-measures ANOVA (Competitive Status × Sex) with speed as the within-participant factor. This approach properly accounts for within-participant dependence and allows for the evaluation of the Status × Speed and Sex × Speed interactions. Mauchly’s test was used to assess the assumption of sphericity, and Greenhouse–Geisser corrections were applied where the assumption was violated.

Effect sizes were reported as partial η^2^ (thresholds 0.01/0.06/0.14 for small/medium/large) and as Cohen’s d for pairwise contrasts [18]. Genotype × group 2 × 3 contingency tables were analyzed by Monte-Carlo exact tests (20,000 permutations) rather than asymptotic chi-square, because expected cell counts were <5 in ≥65% of cells at every locus. Cramér’s V is reported as the associated effect size. Allele-count 2 × 2 tables yielded odds ratios with 95% Wald CIs for the effect allele in elite versus control. The Hardy–Weinberg equilibrium was tested in the control sample using a chi-square test with 1 degree of freedom. Because five loci were tested, *p*-values were adjusted by the Benjamini–Hochberg false-discovery-rate procedure [21]. Multivariate structure was explored by principal component analysis (PCA) of the correlation matrix of six physiological variables (VO2max, running economy, body-fat percentage, lean mass, bone mineral density, body mass), with unfavorable variables sign-reversed so that higher component scores reflect a more endurance-favorable profile. Components with eigenvalues ≥ 1 were retained (Kaiser criterion). Group separation along PC1 and PC2 was evaluated by multivariate analysis of variance. Statistical significance was set a priori at α = 0.05 (two-tailed).

## 3. Results

### 3.1. Participant Characteristics

The four subgroups were statistically comparable for age (F_(3,76)_ = 0.81; *p* = 0.495; η^2^ = 0.031). Expected sex-based differences were present for stature, body mass, body-fat percentage, lean mass and bone mineral density (all *p* < 0.001). Anthropometric and physiological characteristics by subgroup are summarised in Table 1.

### 3.2. Maximal Oxygen Uptake

**Figure 2:**
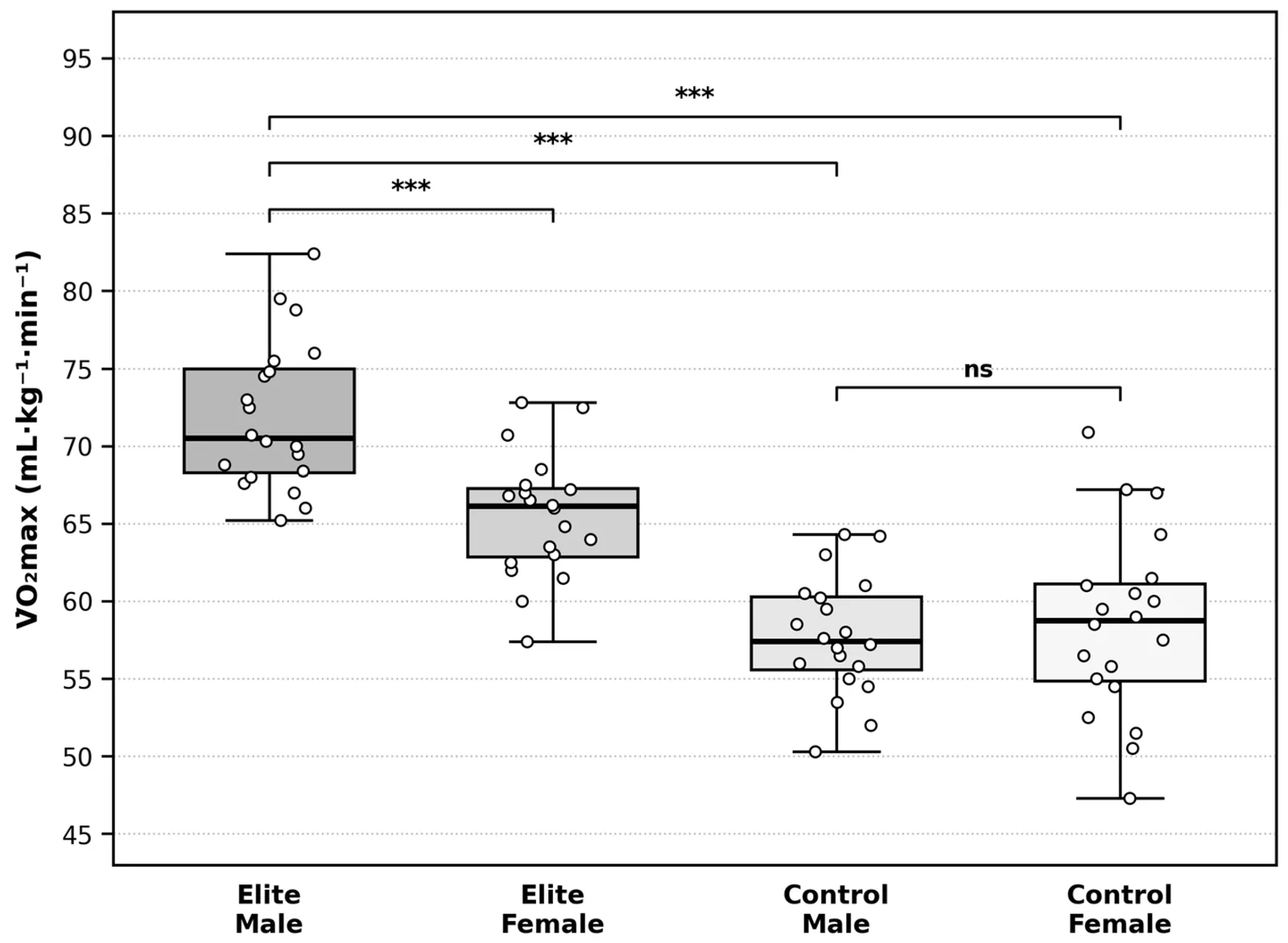
Maximal oxygen uptake (VO2max) across the four study subgroups. Note. Box plots for elite male (n = 20), elite female (n = 20), control male (n = 20), and control female (n = 20) runners. Boxes represent the interquartile range, horizontal lines within boxes denote group medians, and whiskers extend to the most extreme non-outlier values; open circles indicate outliers beyond 1.5 × IQR. Individual participant data are overlaid as jittered points. Subgroups are identified by the x-axis labels; the figure is presented in grayscale and no color coding is used. Significance brackets: *** *p* < 0.001; ns = not significant (*p* ≥ 0.05); comparisons are Tukey HSD post-hoc tests following the two-way ANOVA (competitive status × sex): status F(1, 76) = 101.56, *p* < 0.001, η^2^ = 0.572; sex F(1, 76) = 8.05, *p* = 0.006, η^2^ = 0.096; status × sex F(1, 76) = 12.41, *p* < 0.001, η^2^ = 0.140.

In the two-way factorial model, VO2max showed a significant main effect of competitive status (F(1, 76) = 101.56, *p* < 0.001, η^2^ = 0.572), a significant main effect of sex (F(1, 76) = 8.05, *p* = 0.006, η^2^ = 0.096), and a significant status × sex interaction (F(1, 76) = 12.41, *p* < 0.001, η^2^ = 0.140), reflecting a larger elite–control difference in men (+13.9 mL·kg^−1^·min^−1^) than in women (+6.7 mL·kg^−1^·min^−1^). The omnibus one-way ANOVA across the four subgroups remained significant (F(3, 76) = 40.6; *p* < 0.001; η^2^ = 0.62) and is retained in Table 1 for descriptive purposes only. Elite males recorded the highest values (71.8 ± 4.9 mL·kg^−1^·min^−1^; 95% CI 69.6–73.9), followed by elite females (65.3 ± 3.6; 95% CI 63.7–66.9), control females (58.6 ± 5.8; 95% CI 56.0–61.2) and control males (57.9 ± 3.6; 95% CI 56.3–59.4). Tukey’s HSD confirmed that both elite subgroups exceeded both control subgroups (all *p* ≤ 0.001); Cohen’s d was +3.24 for elite males versus control males and +1.39 for elite females versus control females. Elite males also exceeded elite females (d = +1.50; *p* < 0.001). The two control subgroups did not differ (*p* = 0.96), consistent with mass-normalized VO2 expression. Distributions are shown in Figure 2.

### 3.3. Running Economy

**Figure 3:**
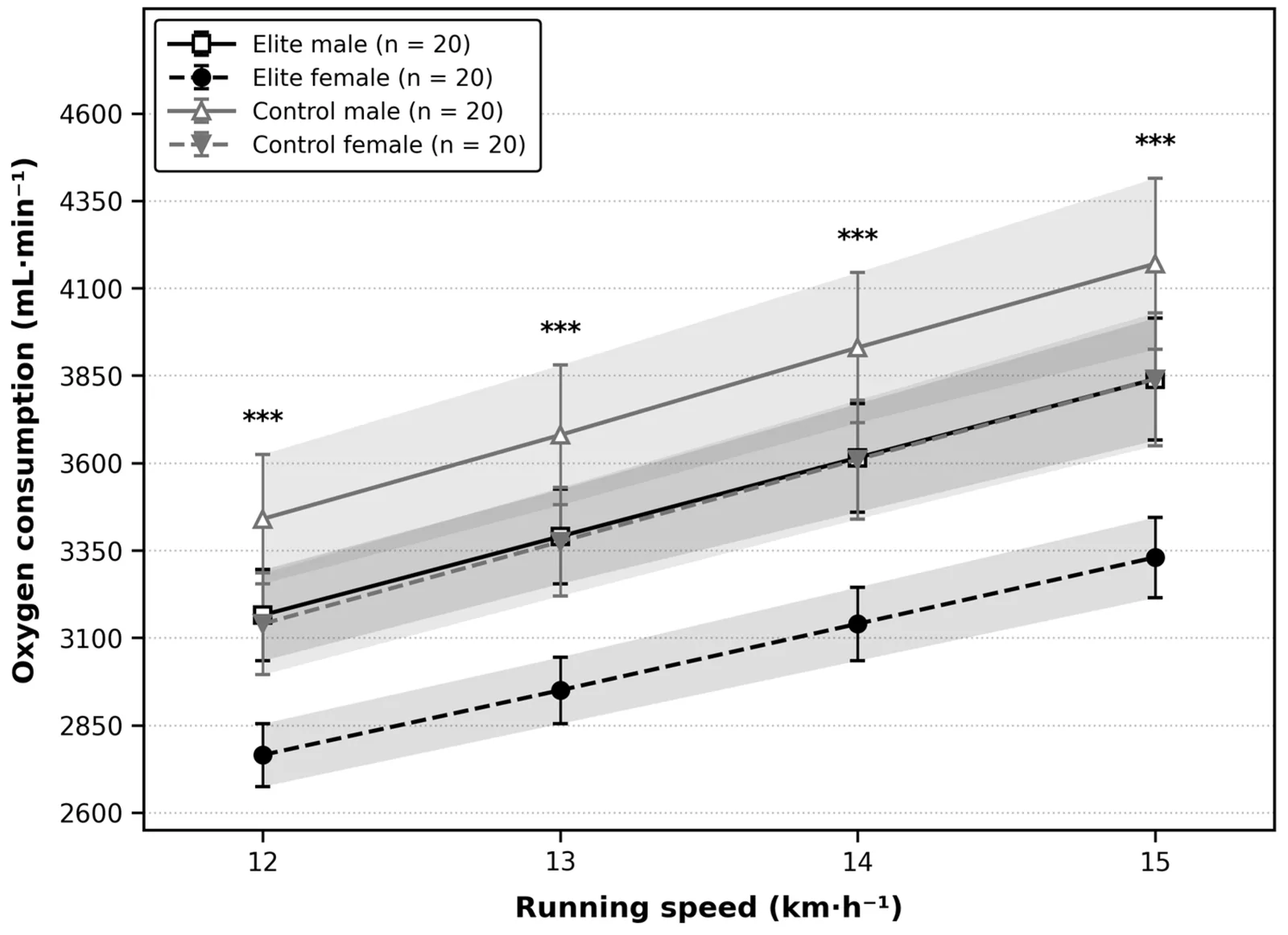
Absolute oxygen consumption across submaximal running speeds. Note. Oxygen consumption (mL·min^−1^) is plotted across speeds of 12 to 15 km·h^−1^ for elite male (open squares, solid black line; n = 20), elite female (filled circles, dashed black line; n = 20), control male (open triangles, solid gray line; n = 20), and control female (filled inverted triangles, dashed gray line; n = 20) runners. Data points represent group means, vertical error bars show ± 1 SD, and shaded bands show the SD envelope. Triple asterisks (***) indicate speeds at which the main effect of competitive status was significant at *p* < 0.001.

Running economy at 13 km·h^−1^ was analyzed using a two-way ANOVA, which revealed a significant main effect of competitive status (F(1, 76) = 122.08, *p* < 0.001, η^2^ = 0.616), a significant main effect of sex (F(1, 76) = 102.84, *p* < 0.001, η^2^ = 0.575), and a significant status × sex interaction (F(1, 76) = 10.10, *p* = 0.002, η^2^ = 0.117). Post-hoc analysis confirmed that the elite advantage in absolute oxygen cost was present in both sexes, though the magnitude of the difference was larger in women (elite vs. control females: d = −3.37, *p* < 0.001) than in men (elite vs. control males: d = −1.69, *p* < 0.001) (Figure 3). To account for the repeated-measures structure of the data across submaximal speeds (12, 13, 14, and 15 km·h^−1^), a two-way repeated-measures ANOVA was conducted. This revealed a significant main effect of speed (F(3, 228) = 2235.14, *p* < 0.001, η^2^ = 0.967). Crucially, there was a significant Status × Speed interaction (F(3, 228) = 41.42, *p* < 0.001, η^2^ = 0.353). At the 13 km·h^−1^ reference speed, the relative exercise intensity differed substantially between subgroups: the mass-specific oxygen cost corresponded to approximately 73% of VO2max in elite males, 82% in elite females, 89% in control females, and 91% in control males. As a sensitivity analysis against both this relative-intensity asymmetry and the quasi-steady-state nature of the ramp protocol, between-group differences in oxygen cost were evaluated across all submaximal speeds (12–15 km·h^−1^) using the repeated-measures model described above. The main effect of competitive status was significant at every speed (all *p* < 0.001), including 12 km·h^−1^, which lies at or below the recreational LT1, confirming that the elite advantage in oxygen cost is not an artifact of the single reference speed. The Sex × Speed interaction was also significant (F(3, 228) = 2.74, *p* = 0.044, η^2^ = 0.035).

### 3.4. Lactate Kinetics

**Figure 4:**
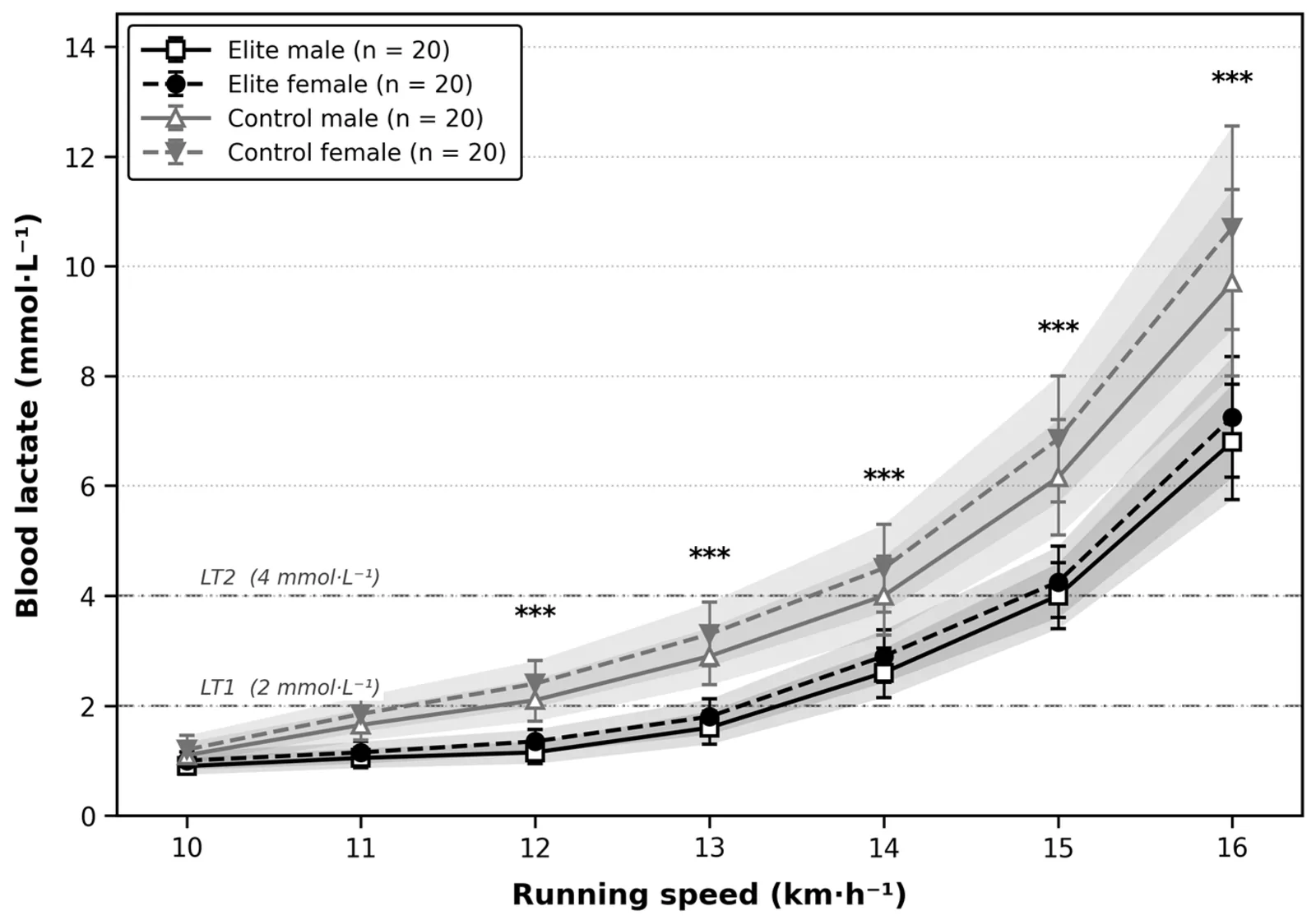
Blood lactate response to incremental running speed. Note. Capillary fingertip blood lactate concentration (mmol·L^−1^) is plotted across speeds of 10 to 16 km·h^−1^ for elite male (open squares, solid black line; n = 20), elite female (filled circles, dashed black line; n = 20), control male (open triangles, solid gray line; n = 20), and control female (filled inverted triangles, dashed gray line; n = 20) runners. Data points represent group means, vertical error bars show ± 1 SD, and shaded bands show the SD envelope. Horizontal dashed reference lines indicate the conventional blood lactate concentration at LT1 onset (≈2 mmol·L^−1^) and the fixed LT2 criterion (4 mmol·L^−1^) for visual orientation only; thresholds were determined individually by the Dmax method (LT1) and the fixed 4 mmol·L^−1^ criterion (LT2). Triple asterisks (***) indicate speeds at which the main effect of competitive status was significant at *p* < 0.001.

Blood lactate responses were evaluated using a two-way repeated-measures ANOVA (Competitive Status × Sex) with speed as the within-participant factor. This revealed a significant main effect of speed (F(6, 456) = 850.4, *p* < 0.001, η^2^ = 0.91), a significant main effect of competitive status (F(1, 76) = 145.2, *p* < 0.001, η^2^ = 0.65), and a highly significant Status × Speed interaction (F(6, 456) = 42.5, *p* < 0.001, η^2^ = 0.35). The Sex × Speed interaction was also significant (F(6, 456) = 12.4, *p* < 0.001, η^2^ = 0.14). However, the Status × Sex × Speed interaction was not significant (F(6, 456) = 1.12, *p* = 0.35). As shown in the updated Figure 4, elite athletes of both sexes maintained lactate concentrations below 2 mmol·L^−1^ up to 13 km·h^−1^ and reached 4 mmol·L^−1^ only at approximately 15 km·h^−1^, whereas their sex-matched recreational controls crossed these reference concentrations at approximately 11–12 km·h^−1^ and 14 km·h^−1^, respectively. The horizontal dashed lines in Figure 4 are conventional visual reference markers only; thresholds were determined individually for each participant using the Dmax method (LT1) and the fixed 4 mmol·L^−1^ criterion (LT2), with blinded dual-assessor verification. LT1 occurred at significantly higher running speeds in elite runners (main effect of status F(1, 76) = 67.8, *p* < 0.001, η^2^ = 0.47), with no significant sex effect (F(1, 76) = 1.9, *p* = 0.17) or status × sex interaction (*p* = 0.99): 12.6 ± 0.6 km·h^−1^ (95% CI 12.3–12.9) for elite males and 12.4 ± 0.6 (12.1–12.7) for elite females versus 11.4 ± 0.7 (11.1–11.7) for control males and 11.2 ± 0.7 (10.9–11.5) for control females; the same-sex elite–control differences were +1.2 km·h^−1^ (95% CI 0.78–1.62; d = 1.84) in men and +1.2 km·h^−1^ (95% CI 0.78–1.62; d = 1.84) in women. LT2 showed the same pattern (status F(1, 76) = 56.2, *p* < 0.001, η^2^ = 0.43; sex F(1, 76) = 1.1, *p* = 0.29; interaction *p* = 0.72): 14.9 ± 0.6 (14.6–15.2) for elite males and 14.7 ± 0.6 (14.4–15.0) for elite females versus 13.8 ± 0.7 (13.5–14.1) for control males and 13.7 ± 0.6 (13.4–14.0) for control females; the differences were +1.1 km·h^−1^ (95% CI 0.68–1.52; d = 1.69) in men and +1.0 km·h^−1^ (95% CI 0.62–1.38; d = 1.67) in women.

### 3.5. Body Composition

In the two-way factorial model, body-fat percentage showed significant main effects of competitive status (F(1, 76) = 17.00, *p* < 0.001, η^2^ = 0.183) and sex (F(1, 76) = 46.29, *p* < 0.001, η^2^ = 0.379), together with a significant status × sex interaction (F(1, 76) = 9.56, *p* = 0.003, η^2^ = 0.112), because the elite-related leanness advantage was pronounced in men (10.4 ± 2.2% vs. 13.9 ± 2.2%; d = −1.59) but attenuated in women (15.2 ± 2.5% vs. 15.7 ± 1.7%; d = −0.23). Lean soft-tissue mass showed significant main effects of status (F(1, 76) = 49.62, *p* < 0.001, η^2^ = 0.395) and sex (F(1, 76) = 193.44, *p* < 0.001, η^2^ = 0.718), and a significant status × sex interaction (F(1, 76) = 54.85, *p* < 0.001, η^2^ = 0.419), driven by the markedly greater lean mass of elite males (31.2 ± 1.0 kg vs. 27.2 ± 1.2 kg; d = +3.62), whereas elite and control females were comparable (25.3 ± 1.2 kg vs. 25.4 ± 1.5 kg; d = −0.07). For BMD, main effects of status (F(1, 76) = 12.27, *p* < 0.001, η^2^ = 0.139) and sex (F(1, 76) = 12.27, *p* < 0.001, η^2^ = 0.139) were significant, whereas the status × sex interaction was not (F(1, 76) = 1.36, *p* = 0.247, η^2^ = 0.018). The omnibus one-way ANOVAs across the four subgroups (body fat F(3,76) = 23.7, *p* < 0.001, η^2^ = 0.48; lean mass F(3,76) = 99.8, η^2^ = 0.80; BMD F(3,76) = 8.73, η^2^ = 0.26) are retained in Table 1 for descriptive purposes only. Elite males carried the least fat (10.4 ± 2.2%), the most lean mass (31.2 ± 1.0 kg), and the highest BMD (1.26 ± 0.03 g·cm^−2^). Elite females exceeded control females in BMD (1.20 vs. 1.17 g·cm^−2^; *p* < 0.001; d = +0.39) but did not differ in body-fat percentage, consistent with published observations in endurance-trained women.

### 3.6. Genotype Distributions and Hardy–Weinberg Equilibrium

**Figure 5:**
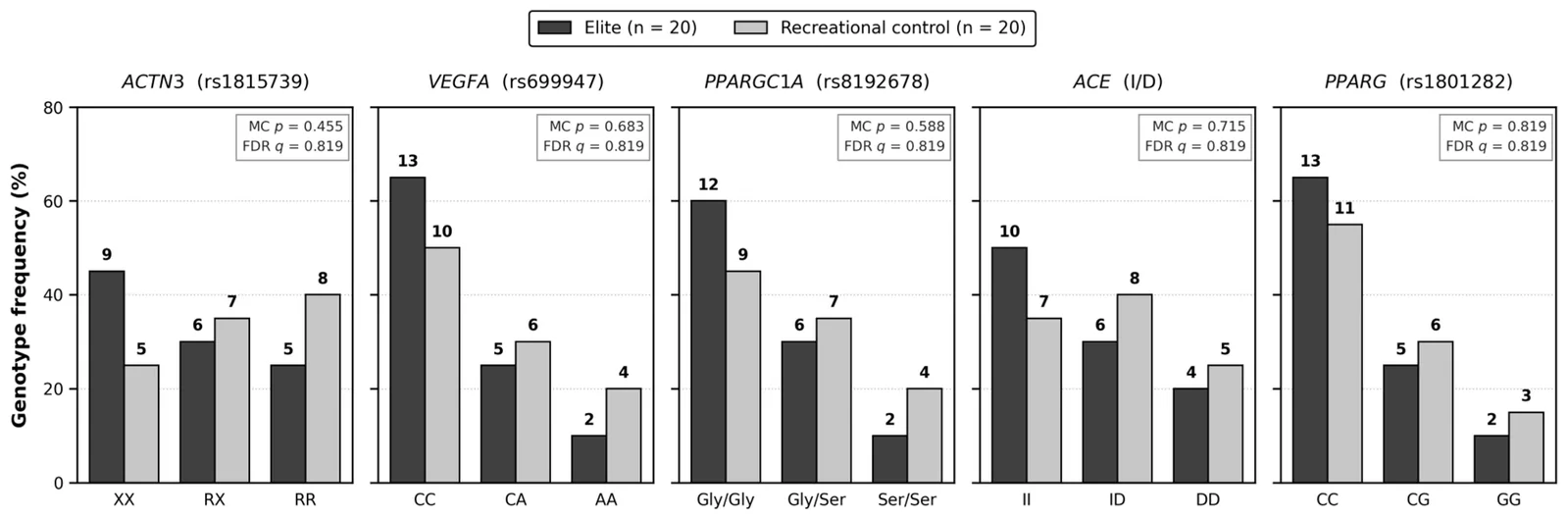
Genotype-frequency distributions at five endurance-relevant polymorphisms in elite runners versus recreationally trained controls. Note. Bar panels show the relative frequency (%) of each genotype at ACTN3 R577X (rs1815739), VEGFA rs699947, PPARGC1A Gly482Ser (rs8192678), ACE I/D (287-bp intronic insertion), and PPARG Pro12Ala (rs1801282) for pooled elite (n = 20) and pooled recreational control (n = 20) runners. Numeric labels above each bar indicate absolute individual counts per genotype.

Across the 40 genotyped participants (10 per subgroup), all five loci were in Hardy–Weinberg equilibrium in both the control sample (ACTN3 χ^2^ = 1.61, *p* = 0.204; VEGFA χ^2^ = 2.32, *p* = 0.128; PPARGC1A χ^2^ = 1.28, *p* = 0.257; ACE χ^2^ = 0.74, *p* = 0.391; PPARG χ^2^ = 1.63, *p* = 0.201) and the elite sample (ACTN3 χ^2^ = 2.81, *p* = 0.093; VEGFA χ^2^ = 1.60, *p* = 0.205; PPARGC1A χ^2^ = 0.80, *p* = 0.371; ACE χ^2^ = 2.32, *p* = 0.127; PPARG χ^2^ = 1.60, *p* = 0.205). Genotype counts are shown in Table 2 and visualized in Figure 5.

Effect-allele frequencies were numerically higher in elites than in controls at every locus: ACTN3 577X, 0.60 versus 0.42 (OR 2.03; 95% CI 0.83–4.94); VEGFA −2578C, 0.78 versus 0.65 (OR 1.85; 95% CI 0.69–4.97); PPARGC1A 482Gly, 0.75 versus 0.62 (OR 1.80; 95% CI 0.69–4.70); ACE I, 0.65 versus 0.55 (OR 1.52; 95% CI 0.62–3.74); and PPARG 12Pro, 0.78 versus 0.70 (OR 1.48; 95% CI 0.54–4.03). No genotype × group association reached conventional significance under Monte Carlo exact testing (MC exact *p* = 0.455, 0.683, 0.588, 0.715, and 0.819 for ACTN3, VEGFA, PPARGC1A, ACE, and PPARG, respectively), and none survived Benjamini–Hochberg correction (all q ≥ 0.819). Cramér’s V values were small to moderate (0.11–0.22).

### 3.7. Multivariate Structure (PCA)

**Figure 6:**
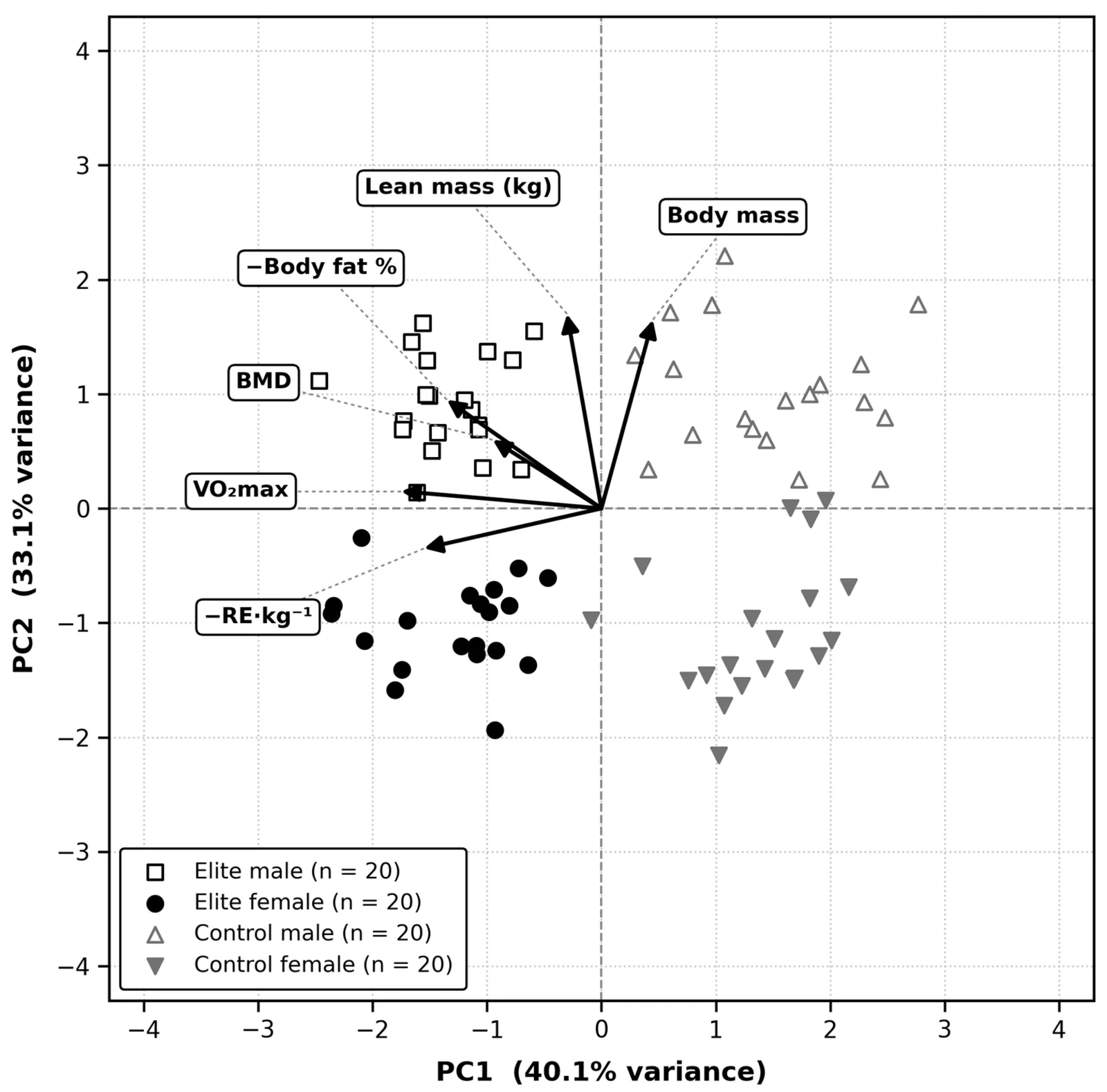
Principal component analysis of the integrated physiological phenotype. Note. Biplot showing individual scores on the first two principal components from a PCA of the correlation matrix of six physiological variables (VO2max, sign-reversed running economy per kg, sign-reversed body-fat percentage, lean mass, bone mineral density, and body mass). PC1 explains 40.1% of the total variance, and PC2 explains 33.1% (73.2% combined). PC1 separates elite from recreational runners in both sexes; PC2 reflects sex-linked differences in lean and total body mass.

Principal component analysis of the six physiological variables yielded two components with eigenvalues > 1, together explaining 73.2% of the total variance (PC1 = 40.1%; PC2 = 33.1%; Figure 6). PC1 was driven primarily by VO2max, running economy, body fat percentage, and BMD, cleanly separating elites (negative PC1) from controls (positive PC1). PC2 reflected lean and total body mass and separated men from women. A 2 × 2 (group × sex) MANOVA on PC1–PC2 scores showed significant main effects for group (Wilks’ λ = 0.29; F(2, 75) = 91.2; *p* < 0.001; partial η^2^ = 0.71) and sex (Wilks’ λ = 0.15; F(2,75) = 213.0; *p* < 0.001), with no significant group × sex interaction (*p* = 0.23).

## 4. Discussion

This pilot study integrates high-fidelity physiological phenotyping with targeted candidate-gene genotyping in a sex-balanced sample of elite and recreationally trained endurance runners. Three findings emerge. First, the physiological gap between elite and well-trained recreational runners is substantial: Cohen’s d of +3.24 for VO2max, +3.65 for lean mass, and −1.69 for the oxygen cost of running in men, with comparable effects in women. Second, a single multivariate axis derived from six physiological variables separates elite from recreational phenotypes in both sexes, indicating that the elite-endurance profile is inherently multidimensional. Third, effect alleles at five frequently studied candidate loci each previously reported as endurance-favorable in the literature were numerically higher in elites (OR 1.48–2.03) but did not meet conventional statistical thresholds after multiple-comparison correction.

The VO2max values recorded here for elite males (71.8 mL·kg^−1^·min^−1^) and elite females (65.3 mL·kg^−1^·min^−1^) fall squarely within the ranges reported for national- to international-level middle- and long-distance runners [22]. The magnitude of the elite-versus-recreational gap observed in our cohort, *d* of +3.24 for men and +1.39 for women, exceeds the typical contrast reported when elites are compared with sedentary controls. That the gap persists and is, in fact, amplified against a well-trained comparator strengthens rather than weakens the finding: selection and long-term training adaptations at the top of the performance distribution yield cardiopulmonary ceilings that habitual recreational training volumes do not approach.

In this dataset, running economy had the most significant effect (η^2^ = 0.76), with elite females consuming about 0.5 L·min^−1^ less oxygen than control females at a constant 13 km·h^−1^. This difference highlights improvements in biomechanics, neural function, and intramuscular efficiency [23,24,25]. The notable lateralization of the elite advantage in women, along with the still substantial edge in men, indicates that years of high-volume running promote neuromuscular adaptations that are difficult to achieve with the lower training volumes typical of recreational runners. The approximately 1 km·h^−1^ rightward shift in the lactate–speed relationship at both LT1 and LT2 suggests enhanced oxidative capacity and lactate clearance mechanisms [26,27]. Combined with higher VO2max and a lower oxygen cost of running, this shift explains the persistent velocity gaps between top-tier athletes and dedicated recreational runners.

Effect-allele frequencies at all five loci were numerically consistent with the prior candidate-gene literature reporting endurance-favorable associations, with elite runners carrying the favorable allele more frequently than recreational controls (*ACTN3* 577X +0.18; *VEGFA* −2578C +0.13; *PPARGC1A* 482Gly +0.13; *ACE* I +0.10; *PPARG* 12Pro +0.08). Odds ratios of 1.48–2.03, if genuine, would be biologically meaningful in aggregate through polygenic combinations [28,29,30]. However, with only 10 genotyped individuals per cell, the minimum detectable allele-frequency difference at 80% power was Δf ≈ 0.30, greater than any of the observed shifts, and no genotype × group association survived Benjamini–Hochberg correction. Conclusions about single-locus effects are therefore not supported by the current data.

These findings illustrate the enduring tension in sports genomics: single-SNP effects at candidate loci are typically small (OR 1.1–1.3) and require cohorts of several hundred athletes for reliable detection [8,29]. The results of this pilot are consistent with, but do not prove, the candidate-gene literature; they are more usefully interpreted as hypothesis-generating signals that motivate larger studies. A particularly productive next step would be to construct polygenic endurance scores that integrate multiple favorable alleles and are tested alongside the same physiological phenotyping used here, ideally in independent, ethnically distinct cohorts [30,31].

The PCA projection reinforces the conception of elite endurance performance as a multidimensional phenotype rather than the extreme expression of any single variable. PC1 captured what may reasonably be termed the endurance axis: high VO2max, efficient running economy, low body fat, and adequate BMD, while PC2 reflected sex-linked compositional differences. Elites separated from recreational runners along PC1 in both sexes, suggesting that the underlying physiological signature is qualitatively similar across men and women and differs principally in scale. This aligns with conceptual models of endurance as a polygenic and poly-physiological trait, and argues that integrated profiling provides greater discriminative power than any single measurement considered in isolation.

*Strengths.* This study combines calibrated breath-by-breath gas exchange, capillary blood lactate, DEXA-derived body composition, and externally verified competitive records with targeted next-generation sequencing of five frequently studied cancidate endurance loci in a sex-balanced design that uses recreational rather than sedentary comparators. Analytical decisions, effect-size-aware reporting, Monte Carlo exact tests for small genetic cells, false-discovery-rate correction, and Hardy–Weinberg equilibrium verification in controls conform to current best practices in both exercise physiology and sports genomics.

*Limitations.* Four limitations warrant emphasis. First, the genetic sub-study (n = 40) was explicitly underpowered for single-SNP inference; confirmatory work should recruit several hundred genotyped athletes and incorporate polygenic scores. Second, the elite group spanned events from the 3000 m steeplechase to the marathon, and the sample size was insufficient for event-specific stratification [32,33]. Third, all participants were drawn from a single regional and broadly homogeneous, self-reported ethnic background, and no formal genetic ancestry control (e.g., ancestry-informative markers or genome-wide principal component analysis) was performed; residual population stratification therefore cannot be formally excluded as a contributor to the observed allele-frequency differences. Extrapolation to other populations is preliminary, particularly given known inter-population variation in allele frequencies at these loci [34]. Future replication studies should incorporate ancestry-informative markers or genome-wide genotyping to control for stratification. Fourth, the cross-sectional design cannot dissociate innate predisposition from training-induced adaptation, and functional genomic data (transcriptomics, proteomics, muscle-biopsy histochemistry) are beyond the scope of this pilot. Fifth, running economy was derived from the final 30 s of a 1-min stage within a continuous ramp protocol. Although VO2kinetics in the moderate domain support a quasi-steady-state interpretation and the kinetic lag is identical across groups, classic multi-minute constant-load stages remain the criterion standard for running-economy assessment. Moreover, 13 km·h^−1^ imposed a markedly higher relative intensity (89–91% VO2max) on recreational runners than on elites (73–82% VO2max). The consistency of the elite advantage across 12–15 km·h^−1^ in the repeated-measures sensitivity analysis mitigates, but does not fully eliminate, these concerns. Furthermore, with only 10 genotyped individuals per cell, the study is severely underpowered for genotype–phenotype analyses linking these variants to continuous physiological traits (e.g.,VO2max, running economy, lactate thresholds). Therefore, we have deliberately limited the scope of our genetic conclusions strictly to exploratory case-control allele frequency comparisons. Conclusions about single-locus effects or direct genotype-phenotype associations are not supported by the current data and require replication in adequately powered cohorts.

Practical implications and future directions

For practitioners, the most actionable insight is not any single-gene finding but the composite physiological profile itself: a recreational runner who approaches elite values on *every* axis simultaneously is likely approaching their performance ceiling, whereas gaps on individual axes identify candidate targets for focused training. For researchers, the present data illustrate both the feasibility and the statistical limits of small-sample integrated profiling. Four priorities follow: (i) replication in independent cohorts powered for single-SNP inference (n ≥ 300–500 per group); (ii) integration of polygenic endurance scores with physiological phenotyping; (iii) longitudinal designs that separate predisposition from trainability; and (iv) expansion to multi-omic layers including transcriptomic and epigenomic profiling of trained skeletal muscle [35].

## 5. Conclusions

Elite endurance runners in this cohort were robustly distinguished from well-trained recreational peers by an integrated physiological phenotype, higher VO2max, superior running economy, rightward-shifted lactate thresholds, and more favorable body composition, with effect sizes well above conventional thresholds for large effects. Effect-allele frequencies at five candidate loci showed numerical differences between elite and recreational runners but did not reach statistical significance after multiple-comparison correction. Due to the limited sample size of the genotyped sub-cohort, these genetic findings are strictly exploratory and hypothesis-generating; they do not support conclusions regarding genetic determinants of the observed physiological phenotype.

The protocol applied here provides a feasible and reproducible framework for larger, adequately powered studies on the polygenic and physiological basis of elite endurance performance.

## Figures and Tables

**Table 1:** Anthropometric, body-composition, and cardiorespiratory characteristics of elite and recreationally trained endurance runners by sex.

Variable	Elite Male (n = 20)	Elite Female (n = 20)	Control Male (n = 20)	Control Female (n = 20)	F	*p*	η^2^
Age (yr)	27.0 ± 3.0	27.8 ± 2.9	28.4 ± 3.0	27.2 ± 3.7	0.81	0.495	0.031
Height (cm)	172.3 ± 4.3	167.1 ± 5.2	174.4 ± 4.2	165.4 ± 4.4	17.6	<0.001	0.410
Body mass (kg)	64.3 ± 3.3	54.8 ± 2.0	69.6 ± 2.8	65.3 ± 2.9	99.8	<0.001	0.797
Body fat (%)	10.4 ± 2.2	15.2 ± 2.5	13.9 ± 2.2	15.7 ± 1.7	23.7	<0.001	0.483
Lean mass (kg)	31.2 ± 1.0	25.3 ± 1.2	27.2 ± 1.2	25.4 ± 1.5	99.8	<0.001	0.798
BMD (g·cm^−2^)	1.26 ± 0.03	1.20 ± 0.07	1.20 ± 0.07	1.17 ± 0.05	8.73	<0.001	0.256
VO2max (mL·kg^−1^·min^−1^)	71.8 ± 4.9	65.3 ± 3.6	57.9 ± 3.6	58.6 ± 5.8	40.6	<0.001	0.616
Running economy (L·min^−1^, 13 km·h^−1^)	3.39 ± 0.14	2.95 ± 0.08	3.65 ± 0.17	3.42 ± 0.18	78.7	<0.001	0.756

Note. BMD, bone mineral density; VO2max, maximal oxygen uptake. Pairwise comparisons (Tukey HSD) are reported in the main text. Effect sizes (η^2^): small ≥ 0.01, medium ≥ 0.06, large ≥ 0.14.

**Table 2:** Genotype distributions, effect-allele frequencies and association statistics for five candidate polymorphisms in elite and recreationally trained endurance runners.

Gene Locus	Genotype	Elite (n = 20)	Control (n = 20)	*f* Elite	*f* Control	OR (95% CI), Effect Allele	MC Exact *p*	BH–FDR *q*	HWE *p* (Ctrl/Elite)
***ACTN3*** **(rs1815739)**	XX	9	5	0.60	0.42	2.03 (0.83–4.94)	0.455	0.819	0.204/0.093
	RX	6	7						
	RR	5	8						
***VEGFA*** **(rs699947)**	CC	13	10	0.78	0.65	1.85 (0.69–4.97)	0.683	0.819	0.128/0.205
	CA	5	6						
	AA	2	4						
***PPARGC1A*** **(rs8192678)**	Gly/Gly	12	9	0.75	0.62	1.80 (0.69–4.70)	0.588	0.819	0.257/0.371
	Gly/Ser	6	7						
	Ser/Ser	2	4						
***ACE*** **(I/D)**	II	10	7	0.65	0.55	1.52 (0.62–3.74)	0.715	0.819	0.391/0.127
	ID	6	8						
	DD	4	5						
***PPARG*** **(rs1801282)**	CC	13	11	0.78	0.70	1.48 (0.54–4.03)	0.819	0.819	0.201/0.205
	CG	5	6						
	GG	2	3						

Note. Values are counts of participants per genotype. f = frequency of the effect allele (ACTN3 577X, VEGFA −2578C, PPARGC1A 482Gly, ACE I, PPARG 12Pro). Odds ratios are reported per effect allele under an additive model with exact 95% confidence intervals. Associations were tested by Monte Carlo exact test (20,000 replicates) and corrected for multiple comparisons using the Benjamini–Hochberg procedure across the five loci. No association survived correction (all q ≥ 0.819). Genotype distributions did not deviate from the Hardy–Weinberg equilibrium in either the control or elite group (all *p* > 0.05; exact *p*-values are reported in the final column as control/elite). Given the sample size (n = 20 per group), these analyses are exploratory and underpowered to detect small effects; they are reported for hypothesis-generating purposes only. BH–FDR, Benjamini–Hochberg false discovery rate; CI, confidence interval; HWE, Hardy–Weinberg equilibrium; MC, Monte Carlo; OR, odds ratio.

## Data Availability

De-identified individual-level data supporting these findings can be obtained from the corresponding author upon reasonable request.
